# Effect of heterozygous deletions on phenotypic changes and dosage compensation in *Arabidopsis thaliana*

**DOI:** 10.1038/s41598-025-98141-6

**Published:** 2025-05-13

**Authors:** Takuya Ikoma, Ryo Nishijima, Miho Ikeda, Kotaro Ishii, Asanga Deshappriya Nagalla, Tomoko Abe, Yusuke Kazama

**Affiliations:** 1 Graduate School of Bioscience and Biotechnology, Fukui Prefectural University, 4-1-1 Kenjojima, Matsuoka, Eiheiji-cho, Fukui, 910-1195 Japan; 2 Department of Radiation Measurement and Dose Assessment, Institute for Radiological Science, National Institutes for Quantum Science and Technology, 4-9-1 Anagawa, Inage-ku, Chiba, Chiba 263-8555, Japan; 3 RIKEN Nishina Center, 2-1 Hirosawa, Wako, 351-0198 Saitama Japan

**Keywords:** Heterozygous deletion, Dosage compensation, Heavy-ion beam, *Arabidopsis thaliana*, Gene expression, Mutation, Plant breeding

## Abstract

Heterozygous deletions, which include a large number of genes, are often caused by the induction of mutations. The induction of gene dosage compensation should be considered when assessing the effects of heterozygous deletions on phenotypic changes. This mechanism is known to balance the expression levels of genes with different copy numbers in sex chromosomes, but it is also known to operate in autosomes. In the present study, 12 *Arabidopsis thaliana* BC_1_ mutants with heterozygous deletions were produced by crossing wild-type Col-0 plants with mutants induced by heavy ion beams. The sizes of the deletions ranged from 50.9 kb to 2.03 Mb, and the number of deleted genes ranged from 8 to 92. Nine of the 12 BC_1_ mutants showed phenotypic changes in fresh weight 14 days after cultivation or during the flowering period. RNA-sequencing (RNA-seq) analyses of 14-day-old leaves, 40-day-old leaves, and flower buds showed that dosage compensation did not occur in any stage or tissue tested. These results indicate that heterozygous deletions cause phenotypic changes owing to the absence of dosage compensation.

**Supplementary Information:**

The online version contains supplementary material available at 10.1038/s41598-025-98141-6.

## Introduction

Recent advancements in mutation induction techniques, such as genome editing^1^, heavy-ion beam irradiation^2^, and the Ex-TAQing system^3^, have enabled the generation of large-scale mutations, including translocations and extensive deletions. These technologies often result in heterozygous deletions, primarily to avoid the lethality that arises from the homozygous disruption of essential genes^2,4^. However, the direct consequences of heterozygous deletions, beyond mere survival, remain an important area of inquiry in plants.

Given these advancements in mutation induction technologies, the question of how heterozygous deletions affect gene dosage and phenotypes becomes increasingly important. Heterozygous deletions can lead to an imbalance in gene copy number, which may have implications for both gene expression and phenotypic outcomes. While the concept of gene dosage compensation has been extensively studied, particularly in sex chromosomes in *Drosophila*^5–8^ and in human^9–11^, its occurrence in autosomes and in cases of heterozygous deletions is less well understood. Studies of autosomal gene dosage compensation in plants have primarily focused on maize (*Zea mays*). Given that both monosomy and trisomy can be produced for all chromosomes in maize, this species is considered tolerant to aneuploidy^12^, facilitating the study of autosomal gene dosage compensation using segmental aneuploidy lines, such as segmental duplications or heterozygous deletions^13,14^. Similar studies have been performed on the model plant *Arabidopsis thaliana*. The expression analysis of individual trisomic lines for each of the five chromosomes indicated that some genes underwent dosage compensation^15^. However, in the case of seven segmentally duplicated lines and six heterozygous deletion lines, gene dosage compensation was rarely observed^3,16^.

The imbalance caused by heterozygous deletions can directly impact organisms. For instance, in the budding yeast *Saccharomyces cerevisiae*, dominant phenotypes are observed in 40% of heterozygous loss-of-function mutants of essential genes, a phenomenon known as haploinsufficiency^17^. Similarly, in *Drosophila melanogaster*, small heterozygous deficiencies on the X chromosome lead to dosage effects, although compensation occurs when the entire X chromosome is affected^18^. These findings indicate that even when one allele remains functional, normal physiological function may be disrupted in the absence of dosage compensation. This suggests that dosage effects can arise in cases of small heterozygous mutations. Such phenomena are likely to result in phenotypic variation in plants as well, underscoring the importance of studying heterozygous deletions in plant species.

In this study, we aimed to evaluate the effects of heterozygous deletions, beyond mere survival after heavy-ion irradiation, on gene expression and phenotype in *Arabidopsis thaliana*. We used 12 BC_1_ lines generated by crossing wild-type Col-0 plants with mutants carrying deletions induced by heavy-ion irradiation. By examining the gene expression patterns and phenotypic changes associated with these heterozygous deletions, we aim to gain insight into the role of gene dosage and its broader implications for plant development and breeding.

## Results

### Development of the heterozygous deletion mutants

To investigate the effects of heterozygous deletions on plant morphology and gene expression in *A. thaliana*, BC_1_ mutants were produced by crossing Col-0 plants and their heavy-ion induced mutants, the mutations of which were detected by whole-genome resequencing, followed by a mutation detection pipeline, AMAP^19^ (see Methods). Each BC_1_ mutant was selected by PCR, using primers designed to detect mutant-specific deletions (Table S1). The sizes of the deletions varied from 42.3 Kbp to 2.03 Mbp (Table S2). Genes included in the deletions were identified according to the *Arabidopsis* Information Resource (TAIR: https://www.arabidopsis.org/). Among the BC_1_ mutants, a total of 34 genes were commonly deleted in all three mutants (C200-56-as4, Ar-443-as1(1), and C30-144-as3). Additionally, 34, 69, and 34 gene deletions were shared between C200-56-as4 and Ar-443-as1(1), C200-56-as4 and C30-144-as3, and Ar-443-as1(1) and C30-144-as3, respectively (Table S2). In total, heterozygous deletions encompassed 496 genes, with 148 genes commonly deleted in two or more BC_1_ mutants. Consequently, we investigated the effects of heterozygous deletions on 382 genes (Tables S3-S14).

### Effect of heterozygous deletions on plant morphology

**Fig. 1:**
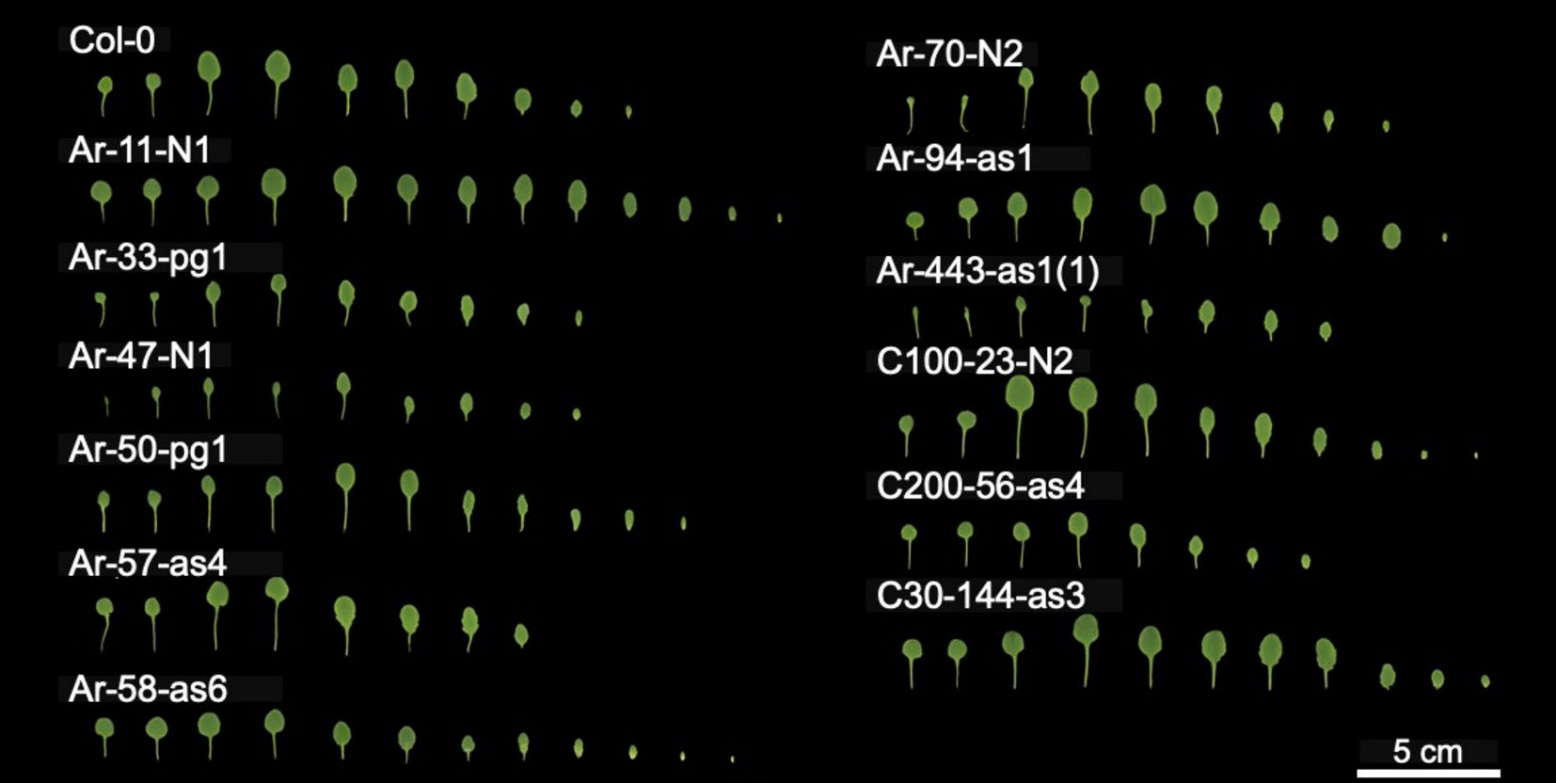
Leaf morphologies of each BC_1_ line. Each mutant was grown in pot at 22.5 °C under long-day conditions (16 h light/8 h dark) in a growth chamber. On 21-day after cultivation started, each leaf was cut and photographed.

**Fig. 2:**
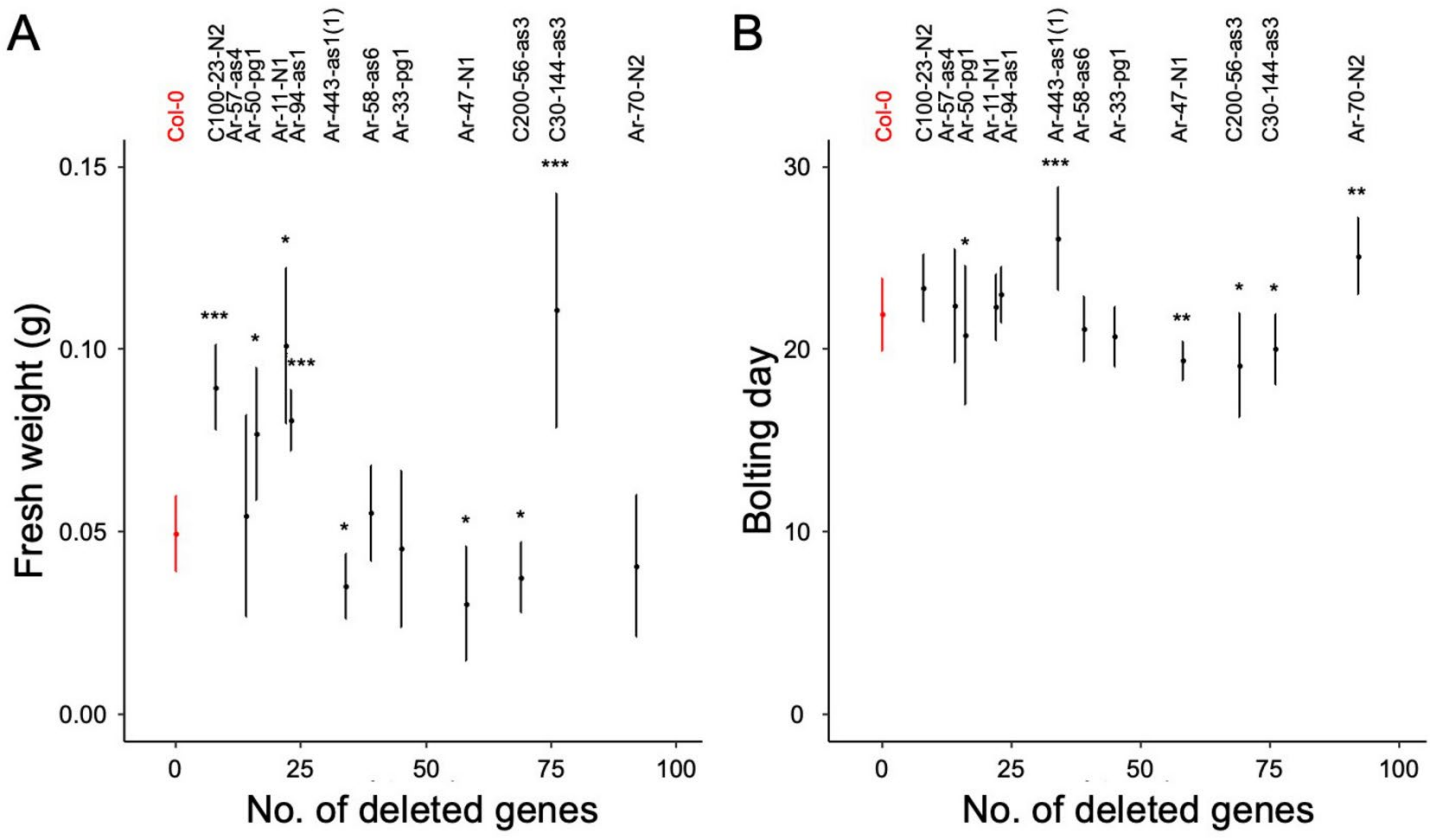
Relationship between the number of deleted genes and fresh weights (**A**) and bolting days (**B**). Red and black plots represent Col-0 and mutants, respectively. Fresh weight and bolting days were measured in at least 6 and 8 plants, respectively. Each line was compared with Col-0 using two-tailed Welch’s *t*-test (**A**) and Wilcoxon’s test (**B**). Statistical significance is indicated as *p < 0.05; **p < 0.01; and ***p < 0.001.

To investigate the effects of heterozygous deletions on plant morphology, we examined leaf shape and fresh weight 21 days after the start of cultivation and flowering time in each BC_1_ mutant. Leaf size and shape differed between the BC_1_ mutants and Col-0 plants (Fig. 1). Fresh weights also varied between BC_1_ mutants and Col-0 plants (Fig. 2a; Table 1); Ar-11-N1, Ar-47-N1, Ar-50-pg1, Ar-94-as1, Ar-443-as1, C100-23-N2, C200-144-as3, and C30-144-as3 showed significantly different fresh weights from Col-0 plants (Fig. 2A; Table S1 and S2, *p* < 0.05, two-tailed Welch’s *t*-test). Specifically, Ar-11-N1, Ar-50-pg1, Ar-94-as1, C100-23-N2, and C200-144-as3 plants weighed more than Col-0 plants, indicating that heterozygous deletions can have beneficial effects on growth in some cases. These effects were independent of the number of deleted genes (Fig. 2; R^2^ = 0.0524).

**Table 1:** Fresh weight and bolting day in each mutant.

Mutant Line	Fresh weight (g)	No. of samples	Bolting day	No. of samples
Ar-11-N1	0.101 (± 0.021)**	6	22.3 (± 1.80)	13
Ar-33-pg1	0.045 (± 0.022)	6	20.7 (± 1.70)	13
Ar-47-N1	0.030 (± 0.016)*	6	19.4 (± 1.10)**	8
Ar-50-pg1	0.077 (± 0.018)*	6	20.8 (± 3.80)*	22
Ar-57-as4	0.054 (± 0.028)	6	22.4 (± 3.10)	13
Ar-58-as6	0.055 (± 0.013)	6	21.1 (± 1.80)	16
Ar-70-N2	0.041 (± 0.019)	6	25.1 (± 2.10)**	9
Ar-94-as1	0.081 (± 0.008)***	6	23.0 (± 1.50)**	7
Ar-443-as1(1)	0.035 (± 0.009)*	6	26.1 (± 2.80)**	13
C100-23-N2	0.090 (± 0.012)***	6	23.4 (± 1.80)	8
C200-56-as4	0.037 (± 0.010)*	7	19.1 (± 2.90)**	8
C30-144-as3	0.111 (± 0.032)***	8	20.0 (± 1.90)**	19
Col-0	0.049 (± 0.010)	7	21.9 (± 2.00)	10

Each line was compared with Col-0, *, *p* < 0.05; **, *p* < 0.01; and ***, *p* < 0.001 (two-tailed Welch’s t-test for fresh weight and Wilcoxon test for bolting day).

Flowering times also varied between BC_1_ mutants and Col-0 plants (Fig. 2b and Table S2); Ar-47-N1, Ar-50-pg1, C200-56-as4, and C30-144-as3 bolted faster than Col-0 plants, whereas Ar-70-N2 and Ar-443-as1 bolted slower than Col-0 plants (*p* < 0.05, two-tailed Wilcoxon rank-sum test). Flowering times were also independent of the number of deleted genes (Fig. 2, R^2^ = 0.0273). Collectively, nine of the 12 BC_1_ mutants tested showed morphological changes, indicating that heterozygous deletions can cause phenotypic changes in *A. thaliana*.

### Effect of heterozygous deletions on gene expression

**Fig. 3:**
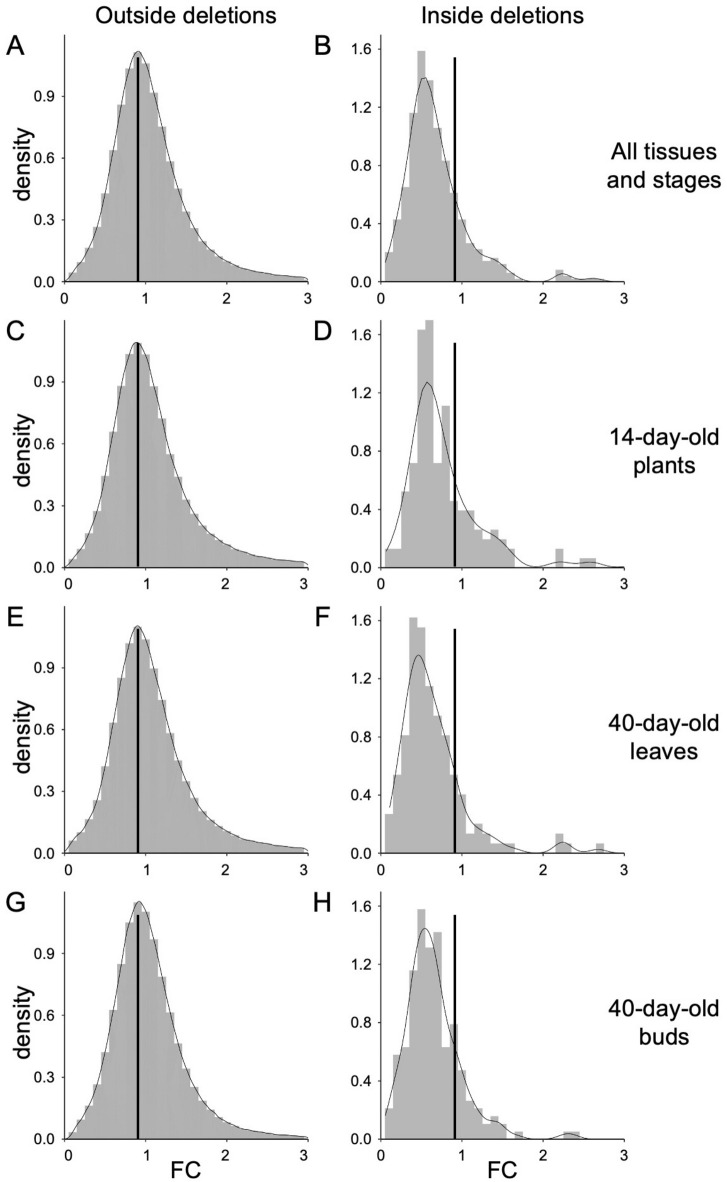
Histograms of gene expression ratios of BC_1_ mutant to Col-0 for genes located outside (**A**,** C**,** E**,** G**) and located inside the deletions (**B**,** D**,** F**,** H**). (**A**) and (**B**): Histograms depicting all samples. (**C**) and (**D**): Histograms depicting plants on day 14 after cultivation started. (**E**) and (**F**): Histograms depicting leaves on day 40 after cultivation started. (**G**) and (**H**): Histograms depicting flower buds on day 40 after cultivation started.

To investigate the effect of heterozygous deletions on gene expression, mRNA was extracted from three independent plants each of the BC_1_ mutant and Col-0, from whole plants 14 d after cultivation started, and from leaves and flower buds 40 d after cultivation started. RNA-seq analysis was performed using a NextSeq 500 (see Methods). When the gene expression ratios of the BC_1_ mutant to Col-0 for each gene in the non-deleted regions were calculated, including all mutant samples, the peak of their density was observed at approximately one, indicating no difference in gene expression levels between the BC_1_ mutants and Col-0 plants (Fig. 3A). By contrast, when the ratios were calculated for genes in the heterozygous deletion regions of all mutant samples, the peak density was observed at around 0.5, indicating that the expression levels of genes in heterozygous deletions were halved (Fig. 3B). The dispersion of densities was significantly different between the non-deleted and deleted regions (*p* < 0.001, Kolmogorov–Smirnov test). Therefore, gene dosage compensation was not observed for most of the genes with heterozygous deletions tested.

The expression levels of genes located within the heterozygous deletion regions in each BC_1_ mutant were investigated and compared with the expression levels of the same genes in Col-0 for each tissue and developmental stage. The results indicated that the median expression levels were significantly reduced in BC_1_ mutants across all tissues and stages, except for whole plants at 14 d post-cultivation, where the difference was not statistically significant (Fig. S1, *p* > 0.05, two-tailed Wilcoxon rank-sum test); although the mutants exhibited lower expression levels. The histogram of the gene expression ratios within the deletion regions of the BC_1_ mutant to Col-0 for each gene also showed peaks at approximately 0.5 (Fig. 3C–H), whereas those outside the region showed peaks at approximately one (*p* < 0.001, Kolmogorov–Smirnov test). These results indicated that gene dosage compensation did not occur in most genes at different developmental stages. However, the histograms showed small peaks around 0.8 in whole plants 14-d after cultivation started and flower buds 40 d after cultivation started (Fig. 3D and H), suggesting the possibility of gene dosage compensation in a small number of genes. The genes undergoing dosage compensation were not strain- or deletion-specific but were scattered across various strains or deletions. Therefore, no deletion specificity is observed in the presence of dosage compensation. When the data were presented in MA plots, increases in expression ratios were observed regardless of the average expression level in all tissues and stages (Fig. S2). Thus, the increase in expression levels in the mutants was not due to the variability in data caused by genes with low expression levels.

**Fig. 4:**
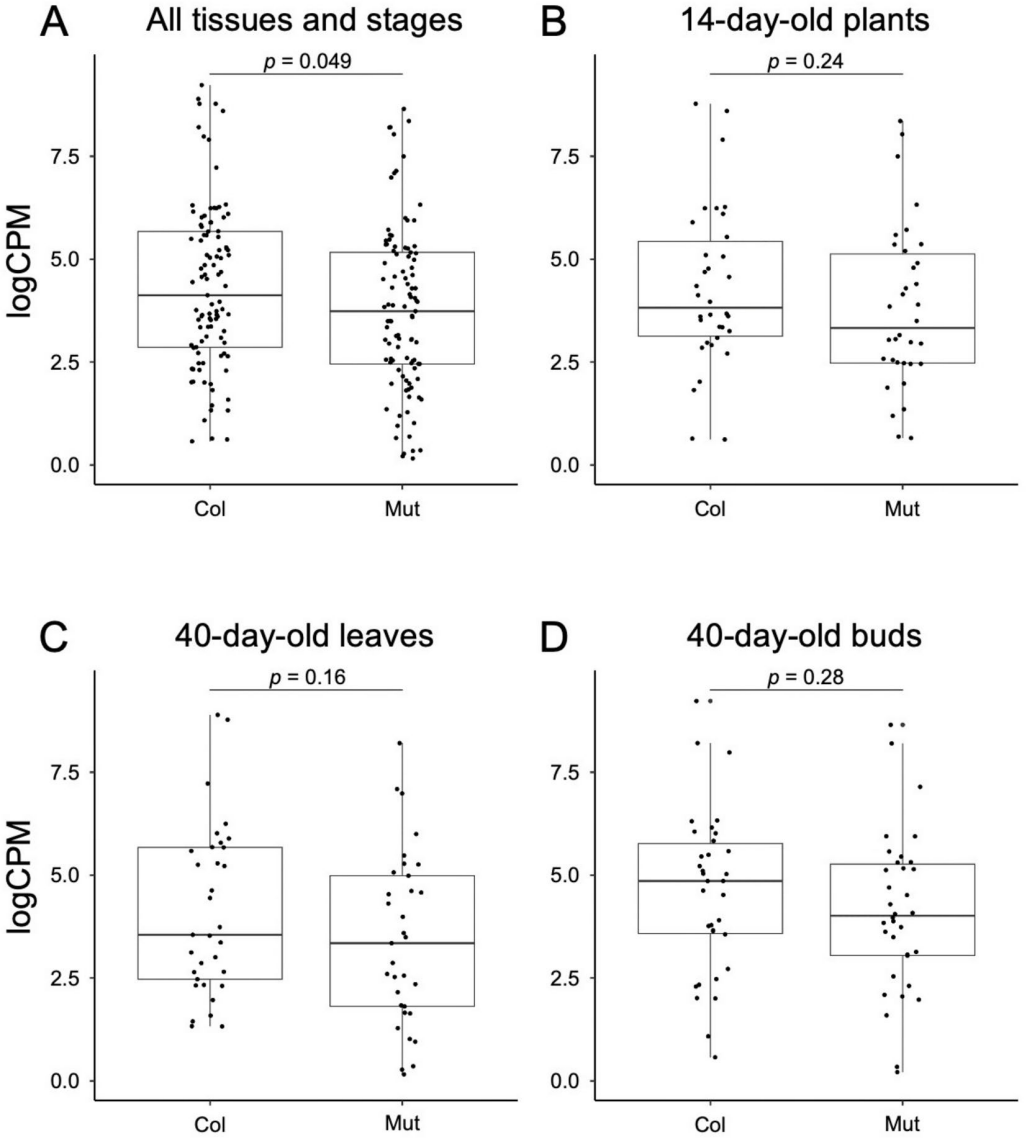
Box-plot comparing Col-0 and the mutant for the expression level of the Dosage Balance Genes (DBGs) inside the deletions in all samples tested (**A**), in plants at day 14 after cultivation started (**B**), in leaves on day 40 after cultivation started (**C**), and in buds on day 40 after cultivation started (**D**). *P*-values of two-tailed Wilcoxon’s test are shown. Box is drawn from first to third quantile with horizontal line showing the median, whiskers indicate minimum and maximum values of the dataset, and each dot represents a gene.

When the copy number of a specific gene is altered, the equilibrium of expression levels between the gene and its partner genes (such as genes encoding subunits of the same complex) is disrupted, resulting in adverse effects on cellular function. These genes are known as Dosage Balance Genes (DBGs)^20^. The DBGs tend to be maintained with their partner genes at dosage balances after whole-genome duplication. Therefore, paralogs that underwent whole-genome duplication were regarded as DBGs in *A. thaliana*^21,22^ (Tables S15–S21). We examined whether dosage compensation occurred in the DBGs included in the heterozygous BC1 deletion mutants. In total, 50 DBGs were included in the heterozygous deletions. A comparison of the expression levels of DBGs in the heterozygous deletions between Col-0 and BC_1_ mutant plants revealed that the median expression levels were significantly lower in the BC_1_ mutants (Fig. 4, *p* < 0.05, two-tailed Wilcoxon rank-sum test). This trend persisted when the median expression levels were examined across three distinct developmental stages. When the expression levels of non-deleted paralogs of heterozygously deleted DBGs were compared between Col-0 plants and BC1 mutants, no significant differences were observed in the median expression levels (Fig. S3, *p* > 0.05, two-tailed Wilcoxon rank-sum test). This result indicates that the upregulation of DBGs does not occur when the copy numbers of their paralog genes are halved.

Ar-443-as1(1), C30-144-as3, and C200-56-as4 exhibited heterozygous gene deletions in the same chromosomal region, which facilitated the detection of consistent upregulation of these genes (Table S22). Notably, AT3G29639 and AT3G29770 showed elevated expression at all sampling stages for both C200-56-as4 and C30-144-as3. AT3G30160 and AT3G30460 showed increased expression exclusively in flower buds and young leaves (14 d after cultivation), respectively. None of these genes encode transcription factors or proteins known to form specific complexes, and their functions remain unidentified. Therefore, a small number of genes may be dose-compensated in a gene-by-gene manner.

## Discussion

Compared to whole-genome gene duplication, heterozygous deletions or segmental duplications may influence the balance of gene expression in the genome, as in the case of X- or Z-linked genes on sex chromosomes. This imbalance can cause phenotypic changes in the absence of gene dosage compensation. In the present study, the effects of heterozygous deletions on phenotypic changes and gene expression levels were investigated using 12 BC_1_ *A. thaliana* mutants produced by crossing Col-0 plants with heavy ion-induced mutants with large deletions.

In *A. thaliana*, dosage compensation does not occur in cases of trisomies, nor in a trans effect in which the expression levels of non-trisomic chromosomes change^15^. BC_1_ mutants tested in the current study did not show gene dosage compensation, corresponding to a previous report in which six different mutants produced by the ExTaqing system were tested^3^. Here, we also demonstrated that dosage compensation did not occur in most of the heterozygously deleted genes across the three developmental stages (Fig. 3). Additionally, our data showed that dosage compensation did not occur in these genes, regardless of their expression levels (Fig. S2). These observations contrast with those in *Drosophila*, wherein gene dosage compensation was observed in genes with high expression ratios in proportion to the average gene expression levels when heterozygous deletions occurred^23^. The reason why gene dosage compensation is absent in *A. thaliana*, despite its similar epigenetic mechanisms to *Drosophila*, remains unclear. In *Drosophila*, autosomal dosage compensation is mediated by change of transcriptome network^24^. Given the presence of the same mechanism in *Arabidopsis*, alterations in expression levels or the number of genes with reduced expression may not significantly impact the transcriptome network. In other words, the deletions analysed in this study may have been too small to trigger gene dosage compensation. In the case of heterozygous deletions, we also observed that the trans-effect did not occur (Fig. 3). The absence of a trans-effect may result from a lack of transcription factors. In our dataset, 22 of 496 genes encoded transcription factors, and no trans effect was observed in any of the BC_1_ mutants (Tables S3–S14). Deletion size is also a possible cause of the absence of trans effects because the number of hemizygous genes is restricted by the deletion size. Indeed, in the case of disomic haploid maize lines where both trans effect and compensation were observed in some extent, in which 876 or more deleted genes were included in individual disomic regions^14^. The number of hemizygous genes is restricted by the deletion sizes. However, the size of deletions induced by heavy-ion irradiation is restricted by the distribution of essential genes in the haploid phase^2^. Taken together, we conclude that gene dosage compensation does not occur in most genes when heterozygous deletions are induced by heavy-ion irradiation in *A. thaliana*.

Gene dosage compensation has been observed in the heteromorphic sex chromosomes of six plant species: *Silene latifolia*^25^, *Cannabis sativa*^26^, *Humulus lupulus*^27^, *Coccinia grandis*^28^, *Rumex hastatulus*^29^, and *Rumex rothschildianus*^30^. This phenomenon likely balances the expression of dosage-sensitive genes, thereby compensating for the degeneration of Y- or W-linked genes. In *S. latifolia*, heavy-ion irradiation-induced large deletions in the Y chromosome led to the upregulation of gene expression levels in X-linked genes homologous to the deleted Y-linked genes, a strict process termed immediate dosage compensation^31^. To explore the evolution of gene dosage compensation in plants, replicating the same experiment on autosomes could serve as a counterpart for the *S. latifolia* study. Our results indicated that immediate dosage compensation does not occur in *A. thaliana* autosomes (Fig. 3). Nonetheless, we found evidence of dosage compensation in some genes (Table S22), implying that these potentially dosage-sensitive genes may contribute to the development of gene-by-gene dosage compensation mechanisms in plant sex chromosomes.

Normally, homozygous genic mutations have been targets of mutation breeding and genome editing, whereas heterozygous deletions have been excluded as the targets due to their lack of full characterization and the impression that they may either have negative effects on plant growth or show no morphological changes. Indeed, some deletions resulted in reduced growth or delayed flowering (Fig. 1; Table 1). However, our current findings indicate that nine of the 12 BC_1_ mutants exhibited morphological changes (Fig. 1; Table 1). Notably, five BC_1_ mutants demonstrated increased fresh weight 14 d after the start of cultivation. These may be due to a direct effect of halved gene expression or halved effect of regulatory elements. These observations suggest that heterozygous deletions could serve as one of genetic resources for breeding despite the challenges in identifying the specific genes responsible for the observed phenotypic alterations. Concerning the use of heterozygous deletions in breeding, further research is needed to investigate their effects on both positive and negative inferences under different environmental conditions as well as in other plant species. Large deletions can be induced by the CRISPR-Cas9 system^32^. Such heterozygous deletions can also be efficiently generated using heavy-ion beam irradiation with high LET values not only in *Arabidopsis*^33,34^ but also in rice^35^ and wheat^36^. Heavy-ion beam irradiation is applied to various tissues, organs, and plant species^37^. Consequently, high-LET heavy-ion irradiation has emerged as a potent tool for mutation breeding by inducing heterozygous deletions, leading to phenotypic variations that may differ from those caused by the complete disruption of a single gene.

## Methods

### Plant materials

*A. thaliana* ecotype Columbia (Col-0), which has been maintained in our laboratory for many years, and mutants derived from it through heavy-ion beam irradiation were used in this study (Table S2). These plants were grown at 22.5 °C under long-day conditions (16 h light/8 h dark) in a growth chamber. Nine of the 12 mutants were previously sequenced, and deletions were detected^2^. An additional three mutants, Ar-50-pg1, C200-56-as4, and C30-144-as3, were produced by Ar-ion beam treatment with a linear energy transfer (LET) of 50 keV µm^− 1^ at a dose of 50 Gy, C-ion beam treatment with an LET of 200 keV µm^− 1^ at a dose of 75 Gy, and C-ion beam treatment with an LET of 30 keV µm^− 1^ at a dose of 400 Gy, respectively. To identify these mutants and their deletions, DNA was extracted from fresh leaves using the NucleoSpin Plant II Mini kit for DNA from plants (Macherey-Nagel, Duren, Germany) and sequenced using the HiSeq 4000 sequencing system (Illumina Inc., https://www.illumina.com) as described previously^34^. The obtained sequence reads were inputted into the mutation detection pipeline AMAP, as described previously, to detect large deletions^19^. From the randomly selected M_2_ plants sequenced, three mutants (Ar-50-pg1, C200-56-as4, and C30-144-as3) were identified to have large deletions. The 12 mutants were crossed with Col-0 plants to produce BC_1_ plants with heterozygous mutations. After crossing, siblings harbouring heterozygous large deletions were selected through genomic PCR on leaves harvested 10 d after cultivation began on 1/2 MS plates containing 1% sugar and 0.7% agar^38^. The primers were designed to detect mutant-specific deletions (Table S1). The resulting BC_1_ mutants, each with heterozygous deletions, were used in subsequent analyses.

### Morphological characterization

The selected BC_1_ mutants were grown with the same ageing Col-0 plants one by one in each pot at 22.5 °C under long-day conditions (16 h light/8 h dark) in a growth chamber. The flowering times of BC_1_ mutants were measured by counting the number of days until bolting. These morphologies were observed 21 d after the start of cultivation. Each leaf was cut and photographed, and fresh weights were measured.

### RNA-seq analysis

Total RNA was extracted from the leaves of the BC_1_ mutants and Col-0 plants 14 and 40 d after cultivation, and from their flower buds 40 d after cultivation using the Nucleospin Plant and Fungi, Mini kit for RNA from plants and fungi (Macherey-Nagel, Düren, Germany). Strand-specific 3-prime mRNA libraries were prepared using BrAD-seq^39^. Briefly, mRNA was isolated from the total RNA using Oligo-d(T)25 Magnetic Beads (New England Biolabs, Ipswich, MA, USA), fragmented by heat and magnesium, and primed for cDNA synthesis using an adapter-containing oligonucleotide. First-strand cDNA was synthesised using RevertAid (Thermo Fisher Scientific, Waltham, MA, USA). The single-stranded portion of a 5-prime adapter was inserted into the breeding terminus of the RNA-cDNA hybrids and incorporated into a complete library molecule using DNA polymerase I (*E. coli*; Takara Bio, Shiga, Japan). PCR enrichment was performed using KOD One PCR Master Mix (TOYOBO, Tokyo, Japan). Libraries were sequenced using an Illumina NovaSeq X Plus 10 B flow cell (Rhelixa Inc., Tokyo, Japan). Only forward reads were mapped to the TAIR10 genome assembly using the STAR aligner with options “--quantMode GeneCounts --outFilterMultimapNmax 1” (version 2.7.10a)^40^, as reverse reads were of low quality due to the poly A sequences. The differentially expressed genes (DEGs) were called glmQLFit in the R package edgeR (version 3.40.2; Robinson et al., 2010)^41^.

## Electronic supplementary material

Below is the link to the electronic supplementary material.

Supplementary Material 1

Supplementary Material 2

## Data Availability

Data were deposited with links to BioProject numbers PRJDB17829 for whole-genome sequencing and PRJDB17834 for RNA-seq of the mutants in the DDBJ BioProject database (https://ddbj.nig.ac.jp/search/en).

## Declarations

### Plant guideline statement

The use of plants in the study were done in accordance with relevant guidelines.
