# Supplementary material for: Effect of heterozygous deletions on phenotypic changes and dosage compensation in Arabidopsis thaliana
Source: Sci Rep. 2025 May 13;15:14284. doi: 10.1038/s41598-025-98141-6 (PMC12075518; doi:10.1038/s41598-025-98141-6)
Supplement: Supplementary file 2 — Supplementary Material 2 [file 41598_2025_98141_MOESM2_ESM.pdf]

Supplemental Figure 1

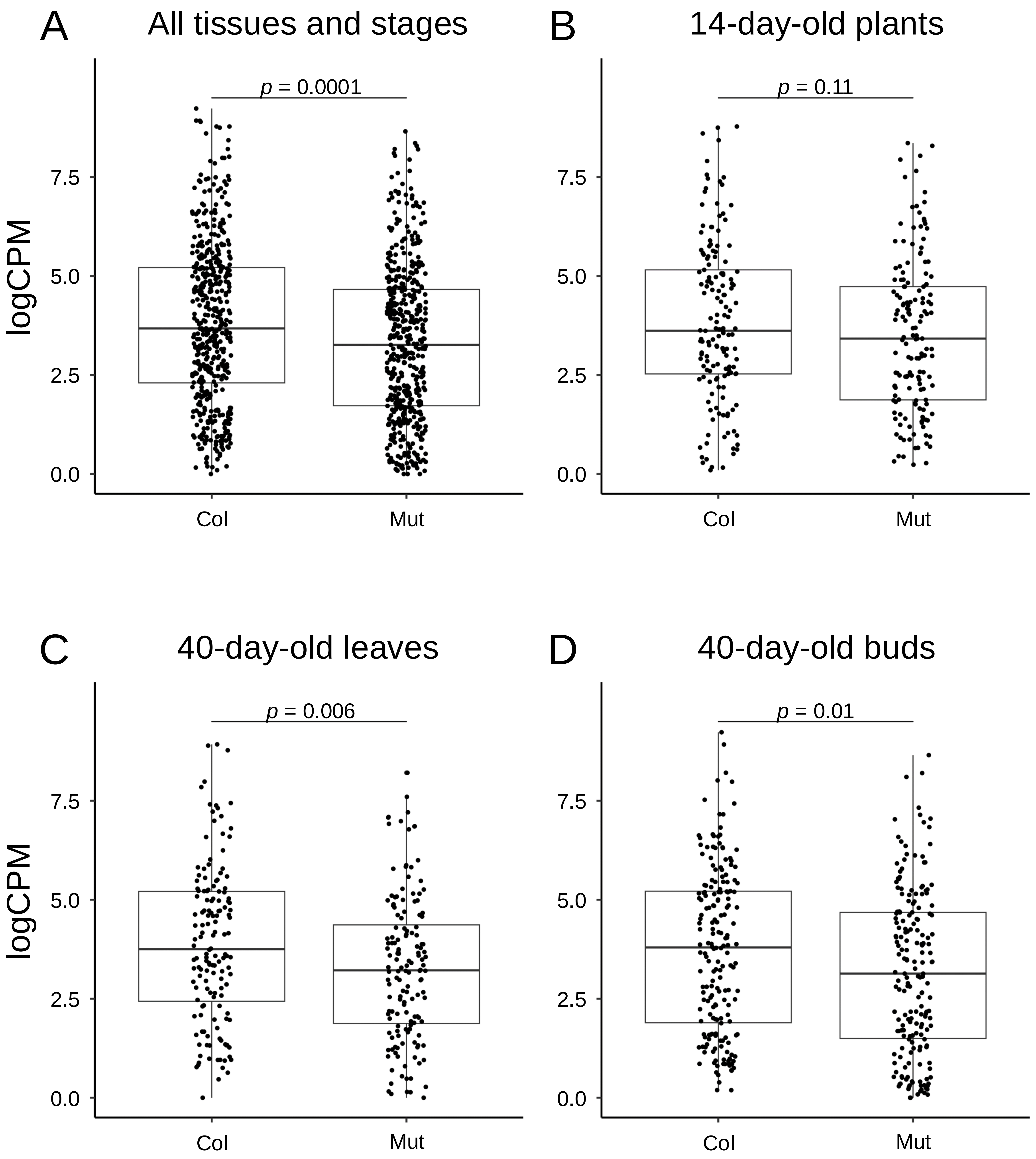

Fig. S1 Comparison of gene expression levels inside the deletions between Col-0 and the mutant for all tissues and stages (A), 14-day-old plants (B), 40-day-old leaves (C), and 40-day-old flower buds (D). Statistical significance is indicated as ***$p < 0.001$, **$p < 0.01$, *$p < 0.05$, and ns, not significant (Welch's *t*-test).

Supplemental Figure 2

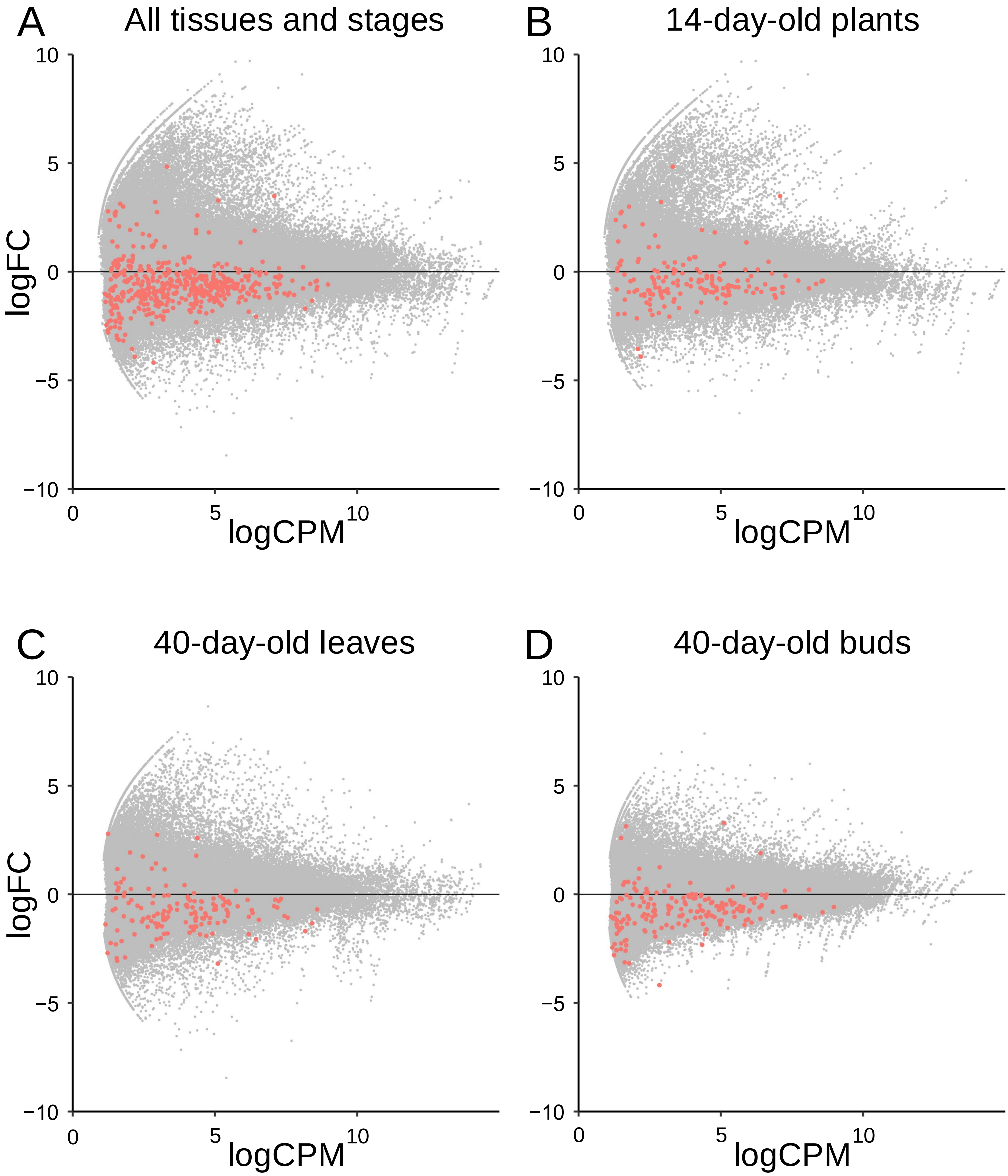

Fig. S2 MA plot with mean gene expression on the horizontal axis and gene expression ratio of mutants to Col-0 on the vertical axis, for all tissues and stages (A), 14-day-old plants (B), 40-day-old leaves (C), and 40-day-old flower buds (D). Red plots indicate intra-deletion genes, and gray plots indicate extra-deletion genes.

Supplemental Figure 3

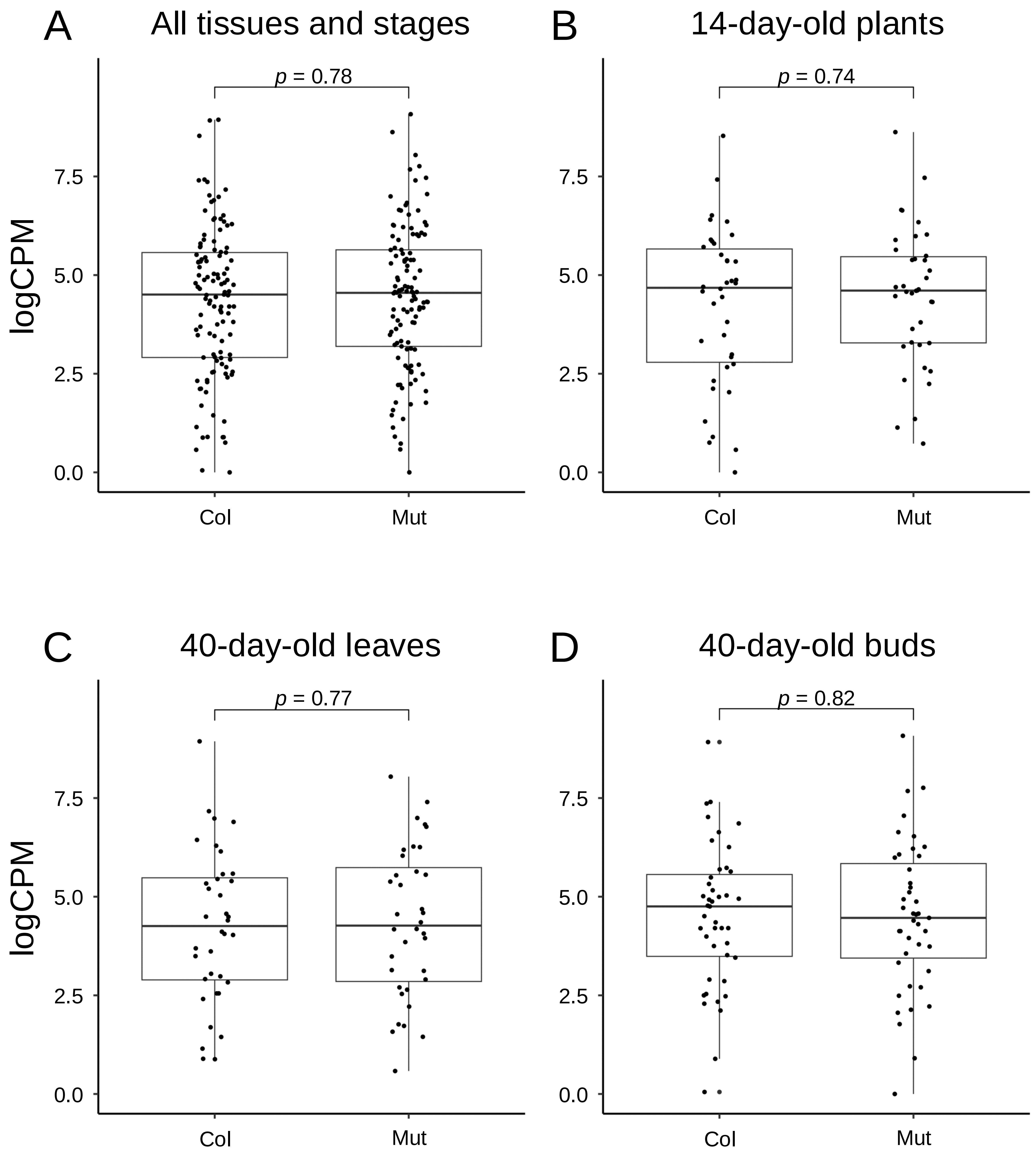

Fig. S3 Comparison of expression levels of DBGs present outside the deletion paired with DBGs inside the deletions between Col-0 and the mutant for all tissues and stages (A), 14-day-old plants (B), 40-day-old leaves (C), and 40-day-old flower buds (D). No significant differences were detected (Welch's *t*-test).
